# Optimization of Plasma Welding Sequence and Performance Verification for a Fork Shaft: A Comparison of Same-Direction and Reverse-Direction Welding

**DOI:** 10.3390/ma18020288

**Published:** 2025-01-10

**Authors:** Jianguang Yang, Peigang Cao, Jiaqing Yao, Junyong Wang, Qilin Mao, Yu Yang

**Affiliations:** 1 College of Mechanical Engineering, North China University of Science and Technology, Tangshan 063210, China; 18332911063@163.com (J.Y.); yjq1176547842@163.com (J.Y.); 2 Tangshan Caofeidian Shiye Port Co., Ltd., Tangshan 063200, China; 3 China MCC22 Group Corporation Ltd., Tangshan 063000, China; fengyuedalu@hotmail.com (J.W.); 13482948403@163.com (Q.M.)

**Keywords:** plasma arc welding, shift fork shaft, welding sequence, numerical analysis, mechanical property

## Abstract

The shift fork shaft is a key component in transmissions, connecting the shift fork in order to adjust the gear engagement. This study investigates the effects of different welding sequences on deformation and residual stress during plasma welding of the shift fork shaft. A temperature-displacement coupled finite element method, using ABAQUS simulation software and a double ellipsoid heat source model, was employed for the numerical analysis. The simulation results show that welding in the same and opposite directions leads to opposite deformation directions but similar deformation magnitudes. However, opposite-direction welding generates more significant stress concentration. After determining an optimal welding process, experimental welding was conducted. Microstructural observations of the weld seam and critical areas, along with mechanical property tests, revealed that the welds were well formed with no surface defects. The heat-affected zone (HAZ) exhibited a mixture of martensitic and non-martensitic phases, while the fusion zone (FZ) underwent phase transformation and recrystallization, forming fine-grained ferrite with martensite. Microhardness (HRC) in the weld seam ranged from 35 to 50, with the FZ and HAZ hardness higher than that of the base material (BM). The second weld pass showed significantly higher hardness in the FZ than the first pass. The tensile strength of the weld joint reached 94% of the base material strength, though plasticity and toughness were reduced. Fracture surface analysis indicated a combination of brittle cleavage and localized plastic deformation.

## 1. Introduction

**Figure 1:**
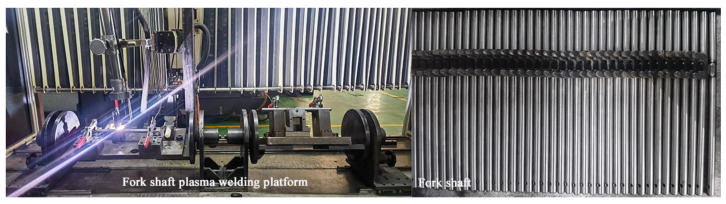
Plasma welding process for a fork shaft.

The shift fork shaft, as a high-precision component in the shifting mechanism, is traditionally manufactured through machining and assembly processes. However, with the advancement of intelligent manufacturing technologies, several innovative manufacturing methods have been proposed to reduce production costs, minimize mold development investments, and meet small-batch demands in the market. One such approach is that of the welding manufacturing process for shift fork shafts, as shown in Figure 1. However, this method must ensure that the product meets the required assembly precision and mechanical performance.

Plasma arc welding (PAW) is widely used in industrial manufacturing due to its high temperature, high energy density, and strong penetration ability, which significantly improve production efficiency and reduce welding distortion. Plasma arc welding can be classified into three methods—penetration-type, fusion-type, and micro-beam-type—depending on the specific requirements of the welding process. Numerous computational and experimental studies by researchers, both domestically and internationally, have proposed various heat source models to describe the thermal characteristics of plasma arc welding. Commonly used heat source models include the combined heat source model, the double ellipsoid heat source model, and the Gaussian surface heat source model. The selection of an appropriate heat source model to ensure accurate thermal analysis and prediction of the welding process depends on the actual morphology of the weld pool during welding [1,2,3].

Wang [4] developed a thermo-elasto-plastic finite element method using MSC software (Version number: 2022) to investigate the impact of welding sequence on residual stress distribution and deformation in butt joints of Q345 steel H-beams. The welding deformations were similar across three different welding sequences. To control residual welding stress, welding the web first is recommended. Jin [5] established a finite element model to optimize the welding sequence for a mixer composed of GH3044 nickel-based superalloy in aircraft engine blades. Simulations demonstrated that single seam symmetric welding is a preferable process, achieving a peak deformation of only 0.54 mm with uniform stress distribution. Barrionuevo [6] evaluated various machine learning regression models, such as Gaussian process, decision tree, and support vector machines, for predicting the ultimate tensile strength (UTS) of AISI 1045 steel and 2017-T4 aluminum alloy joints produced by rotary friction welding with laser assistance. A design of experiments was employed to assess the effects of rotation speed, friction pressure, and laser power on UTS. The results were found to indicate that the gradient boosting regressor (GBR), support vector regressor (SVR), and Gaussian process regressor (GP) outperformed the response surface methodology (RSM) in accuracy, with prediction errors below 3%. Geng [7] conducted a numerical simulation using an axisymmetric coupled thermo-mechanical model to analyze heat transfer and plastic deformation in continuous drive friction welding (CDFW) of 1045 carbon steel and 304 stainless steel. Their results were found to indicate that the heat-affected zone (HAZ) of 304 stainless steel exhibits higher temperatures and lower stresses, with a temperature distribution showing a non-uniform trend from the center to the periphery. The larger deformation of carbon steel is attributed to its lower high-temperature strength. Comparisons between the numerical and experimental results suggest that the developed model can effectively predict temperature and deformation in dissimilar steel welding, while systematically analyzing the effects of welding parameters. Yuan [8] conducted a study using the finite element method (FEM) to investigate welding thermal deformation and welding sequence optimization in order to reduce the residual stress and deformation of copper alloy sheets and improve welding quality. The results show that the trend of residual stress variation in the base material is similar under different welding sequences, and that repeated heating of the same location causes large residual stress. Among the four welding schemes, the sequence that alternates welding from the start and end positions to the middle position resulted in the least deformation, reducing it by 26.6%, 18.3%, and 19.4%, respectively, compared with the other schemes. Mai [9] performed a numerical simulation of the temperature and residual stress distribution in the multi-layer, multi-pass welding process of Weldolet–Header using Simufact Welding finite element analysis software and verified the simulation results through experiments. The results indicate that the welding sequence can change the trend of residual stress distribution. In the first weld pass of the two-stage welding path-2, no high-stress zone was observed. The peak residual stress values for the continuous welding path-1 and two-stage welding path-2 were 428.35 MPa and 434.01 MPa, respectively, with minimal difference. Ding [10] studied the influence of welding sequence and boundary conditions on residual stress and residual deformation in DH36 steel T-joint fillet welds through both experimental and numerical methods. The results show that welding sequence significantly affects the magnitude and distribution of residual stress and deformation. Continuous simultaneous double-sided welding can significantly reduce residual stress and deformation, resulting in higher-quality welds. Boundary conditions have a significant effect on the distribution and magnitude of residual deformation but little effect on residual stress.

In this study, two welding sequence schemes, unidirectional and bidirectional, for the welding operation of shift fork shafts are examined. A numerical model for plasma welding of the shift fork shaft is established to analyze the evolution patterns of the temperature field and the stress–strain field in both welding processes. The aim is to develop effective optimization strategies for the plasma welding process of the shift fork shaft, minimizing the adverse effects of residual stress, enhancing the quality of the welded joints, and ensuring their performance in practical applications.

## 2. Materials and Methods

The shift fork shaft is welded from a fork and trough plate, both made of 45# steel, a typical medium-carbon steel known for its good weldability. However, as the carbon content increases, the weldability decreases. The primary welding challenge lies in the formation of coarse microstructures in both the weld and heat-affected zone (HAZ) under high temperatures, which leads to the development of low-plasticity martensitic structures upon cooling. This results in embrittlement and cracking, significantly reducing the plasticity and toughness of the welded joint, making it a weak point in the entire welded structure [11]. Furthermore, residual stresses generated during welding can further degrade the strength and toughness of the material, meaning that welding performance plays a crucial role in determining the service life of the shift fork shaft [12]. Therefore, it is essential to explore the macroscopic morphology, microstructure, and mechanical properties of the welded joint, especially in the context of plasma welding technology for shift fork shafts. This exploration not only provides insights into the material behavior during welding but also allows for an evaluation of the reliability and durability of the welded joint.

The chemical composition of 45# steel is presented in Table 1. To simplify the computational process, two assumptions are made in the material model: first, the material is assumed to be isotropic and homogeneous; second, when the temperature exceeds the melting point, the yield strength and elastic modulus are assumed to be zero. For the purpose of finite element analysis convergence, these two parameters are assigned small values [13]. During the finite element simulation of welding, the thermophysical properties of the material significantly change with the temperature field. By consulting the data from the “Practical Handbook of Mechanical Engineering Materials” [14] and combining those data with the chemical composition in Table 1, calculations were performed using Jmatpro software(Version number: 7.0.1). This yielded the thermophysical properties of 45# steel at different temperatures, as shown in Table 2 [15,16].

To facilitate the observation of the microstructure of the welded joint, we first used a wire electrical discharge machine to cut the welded joint, obtaining specimens with dimensions of 25 mm × 25 mm × 12 mm. The cut specimens were then embedded in an embedding machine with a hole diameter of 23 mm for the embedding process. Subsequently, different grades of abrasive papers were used to gradually remove scratches from the specimen surface and achieve a smooth surface. The specimens were then polished using a nylon polishing cloth and a 2.5% diamond polishing paste, resulting in a clean and smooth cross-section of the weld. The surface of the specimens was then etched using a 4% nitric acid alcohol solution for approximately 3 to 5 s, until the surface transitioned from a mirror-like finish to a light gray color. Finally, the specimens were rinsed with anhydrous ethanol and dried with a blow dryer, completing the metallographic specimen preparation. The instrument used for observing the microstructure was a JEM-2800F scanning electron microscope (SEM). The equipment used for the tensile test was an MTS Exceed E45 electronic universal testing machine. The tensile test was conducted at room temperature (25 °C) with a testing speed set to 1 mm/min. The penetrant used for the penetrant testing was the HongDa HP-ST penetrant.

**Figure 2:**
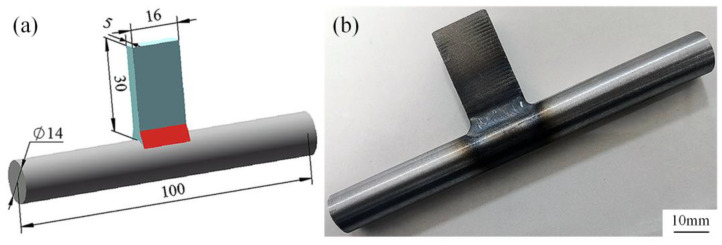
Geometric model and experimental object of welded joints: (**a**) Geometric model (Unit: mm), (**b**) experimental object.

The model includes a trough plate (30 mm × 16 mm × 5 mm), a pin shaft (Φ14 mm × 100 mm) and weld seam (red area in geometric model diagram). The dimensions used are consistent with the actual welding dimensions to simulate the welding process. The geometric model and experimental object of the welded joint are shown in Figure 2.

**Figure 3:**
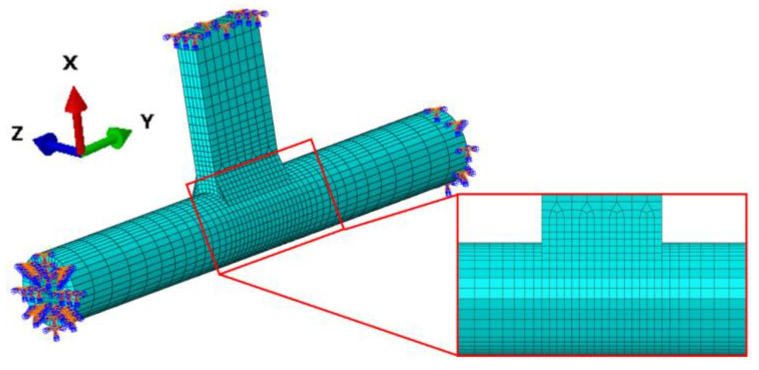
Schematic diagram of finite element model.

The model was partitioned, and hexahedral meshes were generated as shown in Figure 3. To ensure accurate and convergent analysis results, a mesh with a gradient transition from fine to coarse was employed. Specifically, the mesh density in the weld and its surrounding area was set to 1 mm, which was necessary to capture the high gradients of temperature and stress in these critical regions. Gradual mesh transitioning was applied along the axis on both sides of the weld, with a maximum element size of 5 mm, to balance computational efficiency and result accuracy. Additionally, transition meshes with a distal size of 3 mm were used at the junction between the weld and the plate, ensuring smooth integration between the fine and coarse mesh areas. In total, 10,188 elements were used, employing C3D8T elements to simulate the thermomechanical behavior of the welded joint [19,20].

The analysis was conducted in ABAQUS (version number: 6.14.4) using a thermomechanical coupling method. This method was chosen because it allows for the simultaneous simulation of thermal and mechanical behavior, which is essential for accurately modeling the effects of welding on the material. Heat transfer between the welded component and the external environment was considered, with convective boundary conditions applied to all external surfaces. The initial temperature was set to room temperature (20 °C) to reflect the starting conditions. Load constraints were also applied: symmetrical constraints were used on the end faces along the axis of the model to simulate the welding setup, while a fixed constraint was applied to the front-end face of the weld plate to simulate its stationary position during the process [21].

The accuracy of welding temperature field simulations is significantly influenced by the heat source model. The appropriate type of heat source model is generally selected based on the specific welding method, and the heat source parameters are adjusted according to the actual shape of the molten pool to match the real weld boundaries [22]. In this study, penetration-type plasma arc welding with low current was employed for the welding of the fork shaft. The double-ellipsoid heat source model is widely used in thermal simulations of both shallow penetration and deep penetration welds [23]. Therefore, the double-ellipsoid heat source model is more suitable for simulating actual welding conditions.

Adjustments to the double-ellipsoid heat source parameters (Table 3) were made to create an appropriate fusion zone, and the simulated molten pool morphology was compared with the actual weld bead profile on the cross-section of the joint, taking into account dimensions such as weld width, penetration depth, and reinforcement height. The meanings of the heat source parameters in Table 3 are as follows: Q*_f_*/Q*_r_* represents the energy ratio between the front and rear halves of the ellipsoid, *a_f_* refers to the semi-axis length in the welding direction for the front half, *a_r_* refers to the semi-axis length in the welding direction for the rear half, *b* refers to the semi-axis length in the weld width direction, *c* refers to the semi-axis length in the weld depth direction.

**Figure 4:**
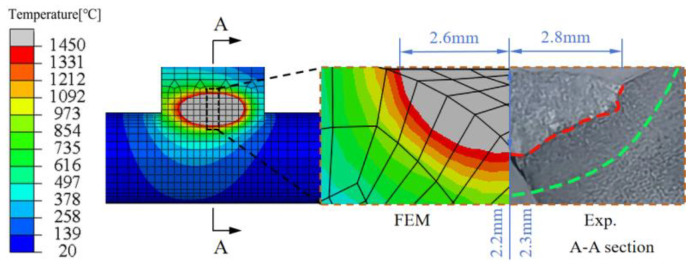
Comparison of finite element simulation and experimental molten pool shapes.

**Figure 5:**
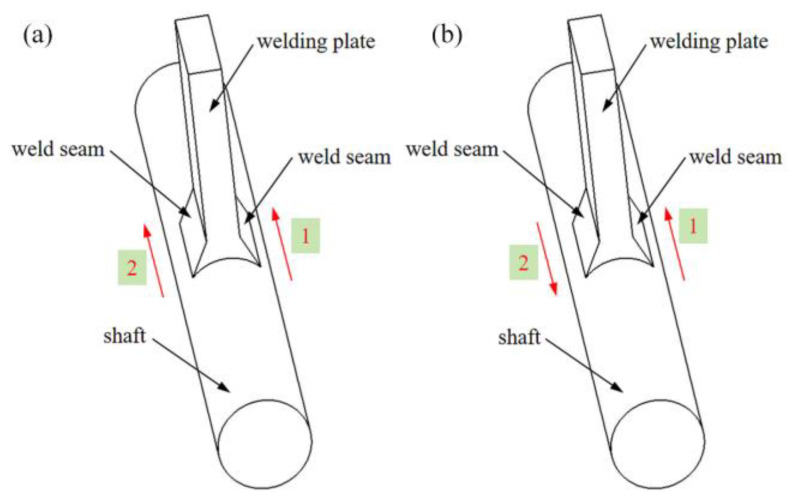
Schematic diagram of welding direction: (**a**) Same-direction welding and (**b**) opposite-direction welding.

Table 4 presents the welding experiment process parameters, and Figure 4 compares the simulated molten pool morphology with the experimental molten pool morphology under the same conditions. It can be observed that the two match well at the same scale, with errors within an acceptable range, thereby validating the accuracy of the plasma arc welding heat source model selected in this study [24,25]. The main difference of the welding sequence scheme is the welding direction of the second weld, which is welded in the same direction and in the opposite direction respectively. The related welding direction is shown in Figure 5.

## 3. Simulation Results and Analysis

### 3.1. Temperature Field of Same-Direction and Opposite-Direction Welding

The weld seam length is 16 mm, with a welding speed of V = 3 mm·s^−1^, resulting in an approximate welding time of 5.33 s for each pass. To more closely approximate actual conditions, the simulation cooling duration was set to 900 s, with the cooling temperature approaching room temperature. In the temperature field study, the heat source positions were selected at t = 1 s and t = 4.5 s for the first weld seam, and at t = 6.5 s and t = 10 s for the second weld seam. The difference in the direction of heat source movement between the two welding methods is primarily observed in the second weld seam. For the first weld seam, the heat source begins at one side of the origin and moves along the seam towards the end. In the case of co-directional welding for the second weld seam, the heat source similarly progresses from the origin to the end. However, in counter-directional welding, the heat source moves in the opposite direction, returning from the end back to the origin.

**Figure 6:**
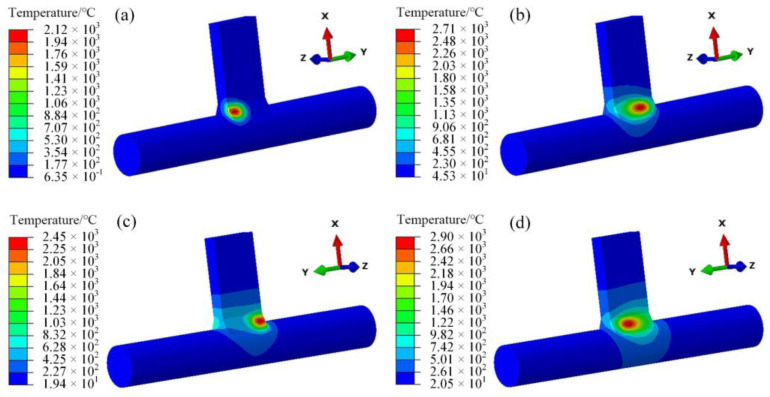
Same-direction welding temperature field: (**a**) t = 1 s, (**b**) t = 4 s, (**c**) t = 6.5 s, (**d**) t = 10 s.

As observed in Figure 6c,d, the temperature profile of the second weld seam differs from that of the first one, with a notably increased width. This is due to the heat conduction within the material induced by the application of the heat source in the first weld seam, resulting in a preheating effect on the second weld seam [26,27].

**Figure 7:**
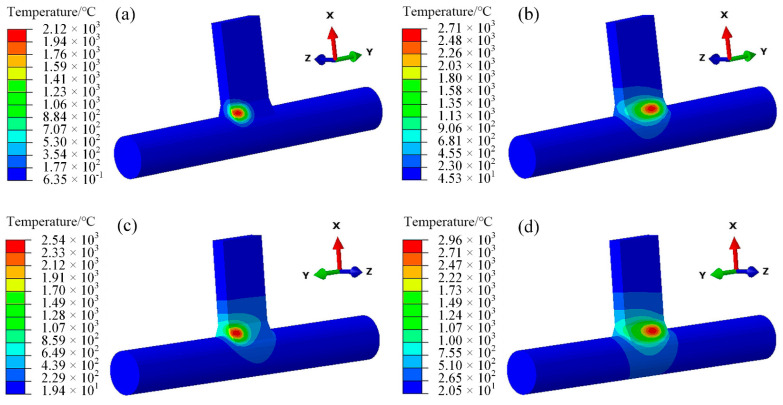
Different direction welding temperature field (**a**) t = 1 s, (**b**) t = 4 s, (**c**) t = 6.5 s, (**d**) t = 10 s. Select three fixed position nodes A, B, and C on the second weld seam, and observe the temperature changes at the same and opposite welding points, as shown in Figure 8.

Comparing (c,d) in Figure 7 and (c,d) in Figure 6, the temperature band of the heat source of the second welding and the same-direction welding is similar, whether it is the starting position of the welding or the intermediate process. However, the peak temperature of the two at the same time is slightly larger than that of the same-direction welding, and there is a difference in the size of the temperature load.

**Figure 8:**
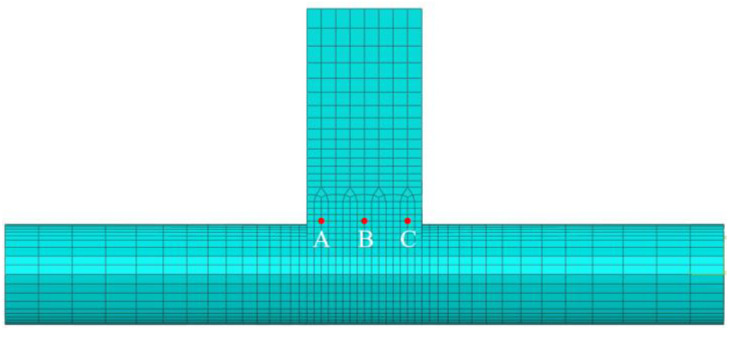
Fixed node selection position.

**Figure 9:**
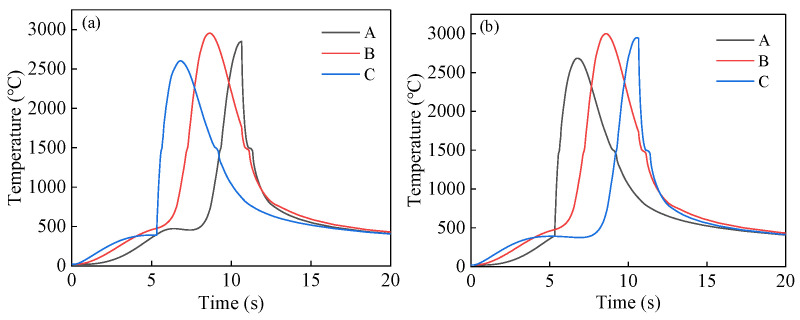
Thermal cycle curve of the node at the fixed position of the second weld seam: (**a**) Same-direction welding and (**b**) opposite-direction welding.

During the second pass of co-directional welding, point A is located at the end of the weld seam, resulting in the peak temperature occurring at a later time. In contrast, during counter-directional welding, the second pass begins heating from the end of the weld seam, with the heat source being continuous between the two weld seams. This causes the peak temperature at point A to appear earlier, while the temperature variation at point C exhibits an inverse pattern to that of point A. As observed from Figure 9, the three nodes undergo two cycles of temperature increase, with the second temperature peak significantly higher than the first. Additionally, the increase in temperature during the second cycle is much steeper compared with the gradual rise in the first cycle. This is because the nodes are located on the second weld seam, where the heat from the first weld seam is conducted to the vicinity of the second weld seam, causing a linear increase in temperature. When the second pass heat source reaches these nodes, the temperature rises rapidly.

### 3.2. Deformation Field of Same-Direction and Opposite-Direction Welding

**Figure 10:**
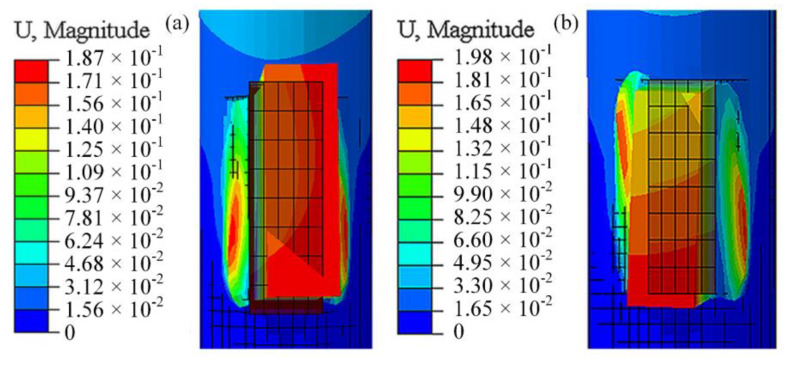
Welding deformation comparison: (**a**) Same-direction welding and (**b**) opposite-direction welding.

By magnifying the welding deformation of the same-direction and opposite-direction welding by a factor of 10, a contour map is generated, showing both the undeformed and deformed regions, with the black boundary representing the pre-weld model, as shown in Figure 10. Through comparative analysis of the deformation location, direction, and magnitude, it can be observed that the deformation is primarily concentrated in the weld seam and weld plate regions, with the red areas being the most prominent. Notably, the deformation direction of the weld plate shows two distinctly different trends, although the maximum deformation difference between the two is only 0.01 mm.

**Figure 11:**
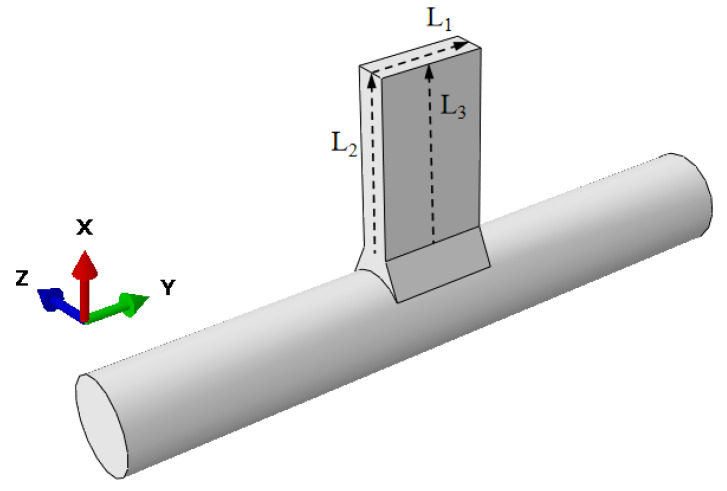
Path setting of deformation.

**Figure 12:**
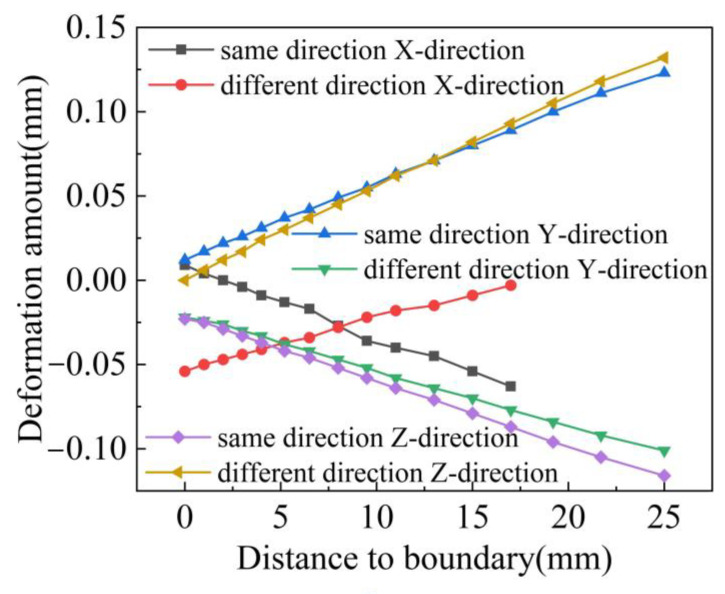
Deformation values in three directions.

To analyze the deformations of the weldment in the *X*, *Y*, and *Z* directions, three paths—L1, L2, and L3—were established, as shown in Figure 11. The deformation values at these three positions were compared, and the results are presented in Figure 12. Observations from Figure 12 reveal that, for both co-directional and counter-directional welding, deformations in the *Y* and *Z* directions are particularly pronounced along paths L2 and L3. On path L2, the *Y*-direction deformation curves show no difference between the two welding approaches. For path L3, the *Z*-direction deformation curve for counter-directional welding exceeds that of co-directional welding, with values ranging from 0 to 0.02 mm. On path L1, the *X*-direction deformation is minimal, and the deformation curves for both welding methods are nearly identical.

### 3.3. Stress Field of Same-Direction and Opposite-Direction Welding

**Figure 13:**
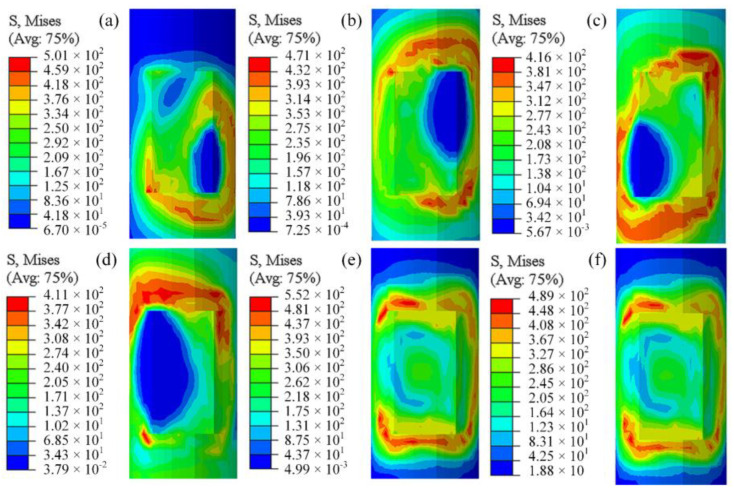
Same-direction welding stress field: (**a**) t = 2 s, (**b**) t = 5.33 s, (**c**) t = 7.33 s, (**d**) t = 10 s, (**e**) t = 40 s, (**f**) t = 800 s.

**Figure 14:**
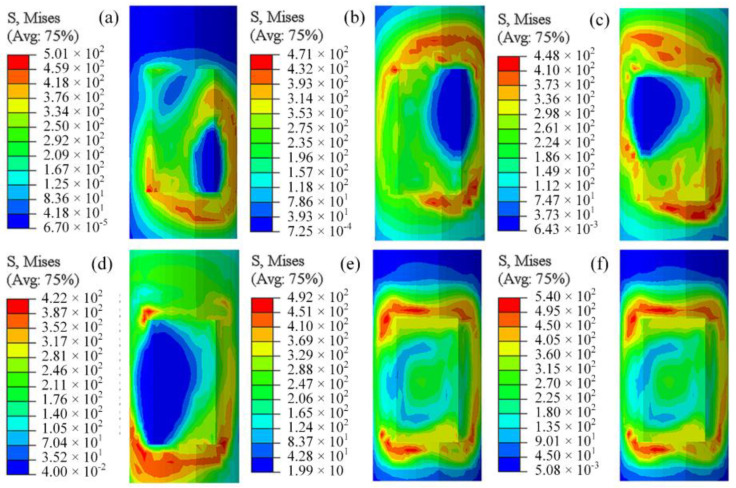
Opposite-direction welding stress field: (**a**) t = 2 s, (**b**) t = 5.33 s, (**c**) t = 7.33 s, (**d**) t = 10 s, (**e**) t = 40 s, (**f**) t = 800 s.

To further understand the evolution of the stress field and analyze the differences in stress distribution between co-directional and counter-directional welding, the stress distribution patterns at different times for both welding approaches were obtained through post-processing, as shown in Figure 13 and Figure 14.

Figure 13a,d reveal that, during the movement of the heat source, stress concentrates around the heat source region, particularly on the side directly exposed to the external environment. After the heat source moves away, the stress values do not rapidly decrease over time. In regions where the heat source energy is concentrated, temperatures are exceptionally high, and the materials are in a molten state with flow occurring, resulting in less stress. It is noteworthy from Figure 13c,d that the heat source from the second weld pass reduces the pre-existing stress, and then new stress distributions form in the areas traversed by the heat source. Figure 13e,f demonstrate that, upon entering the cooling phase, the temperature gradually decreases, which inhibits material movement and increases constraints among the materials, leading to plastic deformation and consequently higher stress values. After 800 s of cooling, the stress at both ends of the weld reaches its maximum value, exceeding the stress observed during the welding process. Due to the influence of the inter-pass temperature, the stress at both ends of the second weld pass is more pronounced than that at the ends of the first weld pass. The overall residual stress exhibits a symmetrical distribution, consistently concentrated around the smaller region surrounding the weld seam during the welding process [28,29].

The stress distribution at different times under the opposite welding scheme is shown in Figure 14, which is not much different from the same-direction welding. The positions of the (c) and (d) stresses in the two welding sequences are obviously different. After the final cooling, the maximum stress of the opposite welding is slightly larger than that of the same direction, and the position is exactly the opposite.

The above contents compare the results of temperature field, deformation field and stress field under two welding sequences, and show the temperature cloud diagram and weld thermal cycle curve at a specific time. At the same time, the deformation and stress evolution process of the three positions of the welding plate in the *X*, *Y* and *Z* directions are analyzed. The results show that different welding sequences have different effects on the deformation of each part. The same-direction welding can reduce the deformation of the weld plate in the Z direction, and the stress area of the weldment caused by the same-direction welding is smaller than that of the opposite-direction welding.

## 4. Macroscopic Morphology and Penetrant Inspection

### 4.1. Macroscopic Morphology

**Figure 15:**
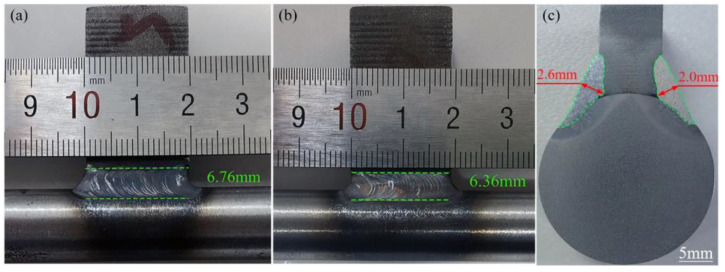
Macro morphology of weld seams: (**a**) First weld, (**b**) second weld, and (**c**) joint cross section.

The welded specimen obtained using the optimized welding technique is shown in Figure 15. It can be observed that the weld seam is well formed with a continuous and smooth surface, exhibiting uniformly distributed fish-scale patterns. No surface defects, such as lack of fusion, undercutting, weld spatter, or noticeable cracks, were observed [30,31]. Measurements indicate that the width of the second weld seam is 0.4 mm narrower than that of the first weld seam. However, the second weld seam exhibits superior smoothness compared with the first, suggesting that the heat transfer from the first weld pass facilitated a more thorough and uniform melting of both the base material and filler metal. As shown in the macroscopic cross-section of the welded joint in Figure 15c, both weld seams achieved full penetration, with no significant welding defects, such as inclusions or porosity, observed. Compared with the first weld seam, the penetration depth of the second weld seam decreased by 0.6 mm, consistent with the change in weld width.

### 4.2. Penetrant Inspection

**Figure 16:**
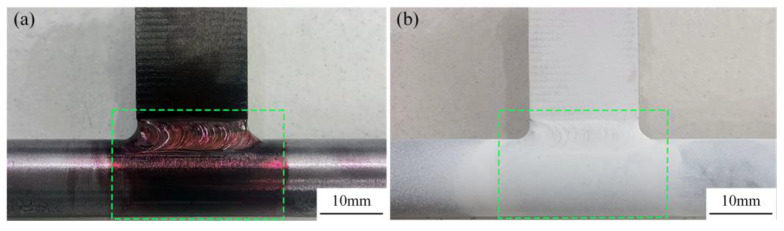
Penetration experiment: (**a**) Spray penetrant agent and (**b**) spray display agent.

The square in Figure 16 shows the surface for weld seam inspection. To perform a penetrant inspection on the weld seam to check for surface cracks, first clean the weld surface. Use a wire brush to remove slag spatter and oxidation scale. Then, use a cleaning agent to thoroughly clean the weld and the area near the weld to remove any dirt, allowing it to air dry. Next, evenly spray the penetrant onto the weld seam, as shown in Figure 16a, ensuring the inspection surface remains wet throughout the entire penetration time, which should be maintained for 30 min. Subsequently, use a cleaning agent and lint-free cloth to wipe the penetrant off the weld seam and allow the surface of the weldment to dry. Finally, shake the developer bottle thoroughly and evenly spray the developer onto the weld seam. Let it sit for 10 min to observe the defect results, as illustrated in Figure 16b. The absence of spot-like and linear indications suggests that there are no pores, inclusions, or cracks on the weld surface [32,33].

### 4.3. Microscopic Morphology

**Figure 17:**
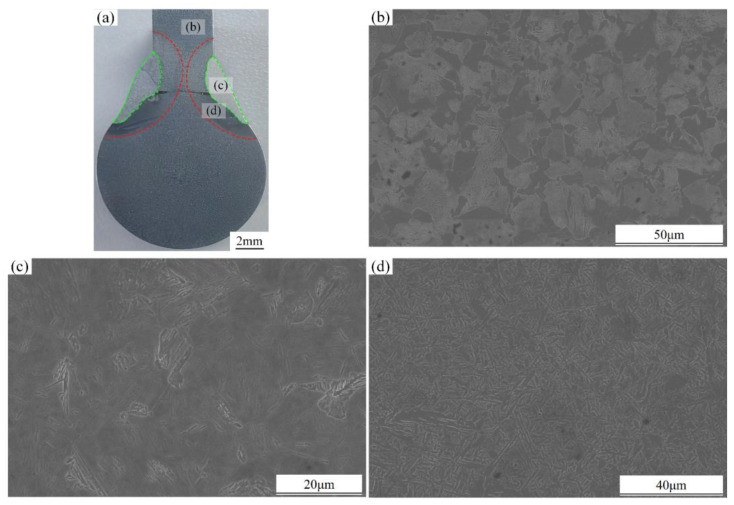
Microscopic structure of different areas of welded joints: (**a**) Welded joints, (**b**) base metal, (**c**) heat-affected zone, and (**d**) fusion zone.

The base material microstructure consists of ferrite and pearlite, with ferrite exhibiting a network distribution along the pearlite grain boundaries, resulting in a uniform structure, as shown in Figure 17b. The heat-affected zone, illustrated in Figure 17c, shows that, due to high temperature exposure, the grains undergo recrystallization, altering the original grain structure and leading to coarsening. Significant microstructural changes occur, forming a mixed structure of needle-like martensite and non-martensitic phases. Figure 17d depicts the weld fusion zone microstructure, which consists of a molten pool formed by the partial melting of the base material and the added welding wire. Upon cooling and solidification, the microstructure undergoes phase transformation and recrystallization, resulting in significant grain refinement [34], producing fine-grained ferrite along with some martensite.

## 5. Mechanical Properties

### 5.1. Microhardness

**Figure 18:**
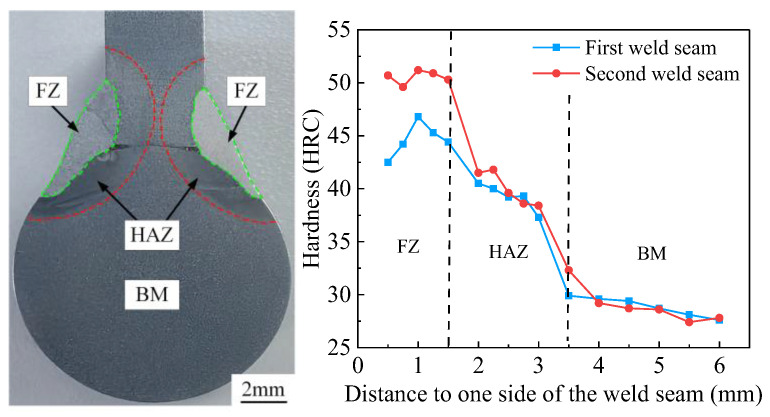
Microhardness distribution in different areas of welded joints.

The microhardness distribution of the two weld seams is shown in Figure 18. Measurements were taken from test points moving sequentially from the weld surface towards the axis center, recording hardness values for the cross-sectional areas of the fusion zone (FZ), heat-affected zone (HAZ), and base material (BM). The hardness exhibits a gradient transition from high to low, moving from the FZ to the BM, which is primarily attributed to microstructural evolution. The hardness of 45# steel BM is approximately 28 HRC. Upon crossing the red line into the HAZ, the hardness increases from 28 HRC to 40 HRC. Moving towards the FZ, passing the green line into the weld zone, the hardness rises to 45–50 HRC. Notably, there are two pronounced increases in microhardness at the interfaces between the BM and HAZ, and between the HAZ and FZ. These increases are mainly due to the thermal cycles experienced by the HAZ during welding, which promote the formation of heterogeneous martensite. In the FZ, the inclusion of low-alloy steel welding wire, along with ferrite and pearlite undergoing phase transformation and recrystallization, leads to grain refinement and is accompanied by fine martensitic structures [35,36,37]. Furthermore, the microhardness of the FZ in the second weld is significantly higher than that in the first weld, indicating that the second weld experienced higher thermal input.

### 5.2. Tensile Properties

**Figure 19:**
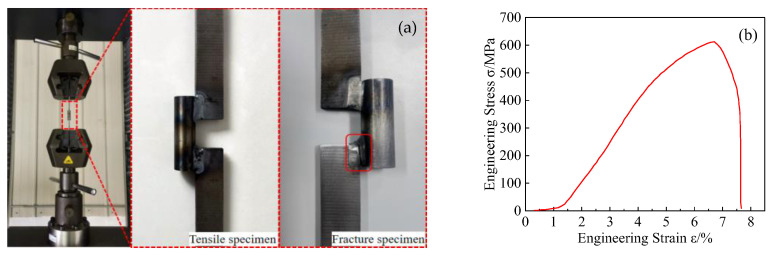
Stress–strain curve of welded joint tensile test: (**a**) Tensile specimen and (**b**) stress–strain curve.

One side of the welded specimen is a pin shaft, while the other side is a trough plate, resulting in an off-center condition. To ensure the representativeness of the tensile specimen, a specially designed tensile specimen was fabricated, as shown in Figure 19a. The weld dimensions and welding processes were kept consistent with the original model. The fracture occurred in the weld zone, indicating that the strength of the welded joint is lower than that of the base material. Figure 19b shows the stress–strain curve for the tensile test of the welded joint, with data indicating that the tensile strength of the welded joint is 612 MPa, which is 94% of the base material’s tensile strength. The yield strength is 315 MPa, and the elongation is 7.7%, which is less than 50% of that of the base material. These results suggest a significant reduction in both the plasticity and toughness of the welded joint compared with the base material (Table 5 [38,39]).

### 5.3. Fracture Morphology

**Figure 20:**
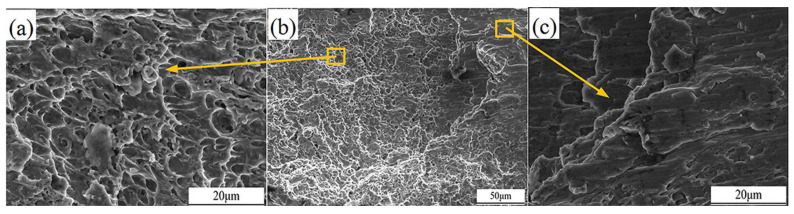
Microscopic morphology of welded joint fracture surface. (**a**) The dimple morphology at the fracture (**b**) Fracture appearance (**c**) River-like morphology at the fracture.

The fracture morphology of the welded joint in tension is shown in Figure 20. The fracture surface exhibits a mixture of cleavage facets and microvoid coalescence features. There are partially smooth and flat areas with pronounced tear ridges, indicating a brittle fracture mode with river-like patterns, as illustrated in Figure 20c. Meanwhile, there are some dimple regions, but the dimples are small in diameter, shallow in depth, sparse in quantity, and unevenly distributed, as shown in Figure 20a. This suggests that, during the fracture process, the weld experienced both brittle cleavage and localized plastic deformation [40].

## 6. Conclusions

(1) The welding sequence significantly influences the distribution of the thermal field during the welding process. In particular, the peak temperature in the reverse welding of the fork shaft exceeds that observed in the direct welding process. Both welding methods induce angular distortion of the plates, though in opposite directions. In the direction of the applied welding force, reverse welding results in a marginally greater angular deformation compared with direct welding. Furthermore, the concentration of residual stress varies with the welding sequence; reverse welding leads to a higher level of residual stress concentration, particularly in localized regions, which can influence the overall structural integrity of the weld.
(2) By carefully selecting the optimal welding sequence and utilizing direct welding, a high-quality weld is achieved, exhibiting excellent surface morphology. The weld is free from common defects such as pores, inclusions, or cracks. Additionally, the second weld pass demonstrates superior flatness compared with the first, with a more complete and uniform melting of both the base metal and the filler material. This improved melting process ensures better fusion and a more stable weld joint, contributing to the overall integrity of the structure.
(3) Detailed microstructural analysis of the heat-affected zone (HAZ) reveals that the welding heat input causes significant grain coarsening, resulting in a mixture of acicular martensite and non-martensitic phases. In the fusion zone (FZ), following the melting and subsequent cooling of the base metal and filler wire, the microstructure undergoes recrystallization, leading to the formation of fine-grained ferrite with a notable presence of martensite. Microhardness testing reveals a distinct hardness gradient from the FZ to the base metal (BM), which corresponds to the microstructural variations across these regions. Notably, the microhardness in the FZ of the second weld pass is significantly higher than that of the first pass, emphasizing the pronounced effect of inter-pass temperature interactions on the final hardness profile.
(4) The tensile strength of the welded joint reaches a peak of 612 MPa, which is 94% of the tensile strength of the base material. However, the elongation of the welded joint is 7.7%, which is only 48% of that of the base material. The fracture mode of the welded joint exhibits a combination of brittle and ductile fracture characteristics. The fracture surface displays both smooth, river-patterned zones, indicative of brittle fracture, as well as dimpled regions, which are typical of ductile fracture. This mixed fracture behavior suggests that, while the weld exhibits high tensile strength, its ductility is reduced compared with the base material.

## Figures and Tables

**Table 1:** The chemical composition of 45# steel (wt.%) [17,18].

C	Si	Mn	S	P	Cr	Ni	Cu
0.42~0.50	0.17~0.37	0.50~0.80	≤0.035	≤0.035	≤0.25	≤0.25	≤0.25

**Table 2:** Change of material thermo-physical parameters with temperature.

Temperature/ °C	Specific Heat/10^6^ (J∙kg^−1^∙K^−1^)	Density/ 10^−9^ t/mm^3^	Thermal Conductivity/ (W∙m^−1^∙K^−1^)	Elastic Modulus/ 10^5^ MPa	Thermal Expansion/10^−5^ °C^−1^	Yield Strength/ MPa
20	465	7.81	54.5	2.06	0	426
300	485		49.5		1.31	310
400	523	7.81	41.45	1.85		
700	854	7.81	31.82	1.44	1.50	273
800	513		25.96	1.30	1.25	128
1000	602	7.81	29	1.08	1.44	70
1200	639		31.3	0.87		20
1400	654	7.81	33.4	0.01		1

**Table 3:** Heat source parameters.

Q*_f_*/Q*_r_*	*a_f_*	*a_r_*	*b*	*c*
0.5	5	10	6	2

**Table 4:** Model validation test process parameters.

Welding Speed/mm·s^−1^	Welding Current/A	Welding Voltage/V
2.5	105	26

**Table 5:** Mechanical performance indicators of 45# steel.

Tensile Strength/MPa	Yield Strength/MPa	Elongation Rate/%
≥600	≥355	≥16

## Data Availability

The original contributions presented in this study are included in the article. Further inquiries can be directed to the corresponding author.
